# Blood Pressure Lowering Effect of Cuban Policosanol is Accompanied by Improvement of Hepatic Inflammation, Lipoprotein Profile, and HDL Quality in Spontaneously Hypertensive Rats

**DOI:** 10.3390/molecules23051080

**Published:** 2018-05-03

**Authors:** Kyung-Hyun Cho, Dhananjay Yadav, Suk-Jeong Kim, Jae-Ryong Kim

**Affiliations:** 1 Department of Medical Biotechnology, Yeungnam University, Gyeongsan 712-749, Korea; dhanyadav16481@gmail.com (D.Y); superiorgene@ynu.ac.kr (S.-J.K); 2 Research Institute of Protein Sensor, Yeungnam University, Gyeongsan 712-749, Korea; 3 LipoLab, Daehak-Ro 280, Gyeongsan 712-749, Korea; 4 Department of Biochemistry and Molecular Biology, Smart-Aging Convergence Research Center, College of Medicine, Yeungnam University, Daegu 705-717, Korea; kimjr000@gmail.com

**Keywords:** spontaneously hypertensive rats, policosanol, lipoproteins, hypertension, hepatic inflammation

## Abstract

We investigated the antihypertensive effect of policosanol on spontaneously hypertensive rats (SHR). For this, we analyzed blood pressure, blood lipid, and lipoprotein properties in male SHR after consumption of Cuban policosanol (PCO). The experimental groups were as follows: normotensive Wistar Kyoto (WKY) control, SHR group fed normal diet (ND), SHR group fed 20 mg of PCO, SHR group fed 100 mg of PCO, and SHR group fed 200 mg of PCO per kg of body weight. After eight weeks, the SHR control group showed gradual increases up to 22% in systolic blood pressure (SBP) and 17.6% in the diastolic blood pressure (DBP) compared with values at week 0. However, policosanol consumption had a dose-dependent reduction effect on SBP and also reduced DBP up to 16% in a dose-dependent manner. Heart rate (HR) bpm increased by six percent in the SHR control, whereas the 20 mg, 100 mg, and 200 mg of policosanol groups showed a reduction of 36%, 28%, and 34% respectively. Although serum total cholesterol (TC) level of SHR was not affected by policosanol consumption (70–80 mg/dL), serum triglyceride (TG) level significantly decreased in the SHR + 200 mg of PCO group. Serum high-density lipoprotein cholesterol (HDL-C) level was also significantly elevated by policosanol consumption. The % HDL-C/TC ratio was elevated in the policosanol group up to 67–70%, whereas the SHR control group showed a ratio of 58%. Serum cholesteryl ester transfer protein (CETP) activity was reduced by policosanol in a dose-dependent manner. Although the serum glutamate oxaloacetate transaminase (GOT)/ glutamate pyruvate transaminase (GPT) were similar across all groups, policosanol consumption caused reduction of reactive oxygen species (ROS) levels in hepatic tissue. The SHR control group showed a 2.1-fold higher serum C-reactive protein (CRP) level than the WKY group, whereas the CRP level decreased in the SHR + 200 mg of PCO group (up to 45%) than SHR control group. Aldosterone level was reduced in the policosanol group (up to 34%) in a dose-dependent manner compared to the control. In conclusion, eight weeks of policosanol consumption in SHR resulted in remarkable reduction of blood pressure, serum aldosterone, and serum TG levels along with the elevation of HDL-C and improvement of hepatic inflammation.

## 1. Introduction

High blood pressure is a global risk factor for coronary artery disease and metabolic syndrome and is associated with high oxidative stress and aortic stiffness. Dyslipidemia and hypertension are hallmarks of metabolic syndrome. New onset hypertension was preceded by dyslipidemia [1]. High triglyceride (TG) and low-density lipoprotein cholesterol (LDL-C) levels along with a low high-density lipoprotein-cholesterol (HDL-C) level are strongly associated with the development of hypertension [1,2].

Oxidative stress is strongly associated with hypertension as an underlying causative factor [3]. Although the role of oxidative stress in the development of hypertension has not been fully elucidated, endothelial dysfunction is deeply involved in hypertension. HDL is a potent antioxidant molecule in the blood, and it can ameliorate endothelial dysfunction to exert vasoprotective functions. It is well known that HDL mediates many pleiotropic functions to protect against cardiovascular disease and hypertension and possesses cholesterol efflux and NO production activities [4].

The development of a new agent that can simultaneously lower blood pressure and improve the lipid profile for treatment of cardiovascular disease (CVD) and hypertension is a major research goal. However, until now, there has been no promising pharmaceutical or nutraceutical to treat hypertension and hyperlipidemia simultaneously.

It has been previously reported that policosanol is effective in reducing the risk of CVD raising HDL-C and lowering LDL-C levels. A recent meta-analysis of randomized controlled trials from 22 studies including 1886 subjects concluded that policosanol could significantly reduce TC and LDL-C as well as increase HDL-C levels [5].

Encapsulation of policosanol by reconstituted HDL (policosanol-rHDL) was reported to enhance anti-oxidant and anti-glycation activities, resulting in inhibition of cellular senescence. Policosanol-rHDL (final concentration of nine micromolars of policosanol) showed more potent antioxidant activity than vitamin C (final 100 µM) in a previous study [6]. In a hypercholesterolemic zebrafish model, policosanol supplementation for nine weeks resulted in lowering of serum TC and TG levels and elevation of HDL-C via CETP inhibition, and amelioration of fatty liver changes and reactive oxygen species (ROS) production [7]. Although the exact mechanism of policosanol on the lowering of lipid levels and blood pressure has not been fully identified; however, a few of studies have evaluated that policosanol involves various pathways such as down-regulation of HMG-CoA reductase [8], CETP inhibition [9,10] and renin–angiotensin–aldosterone system that might help in reducing lipid and blood pressure level [11]. However, the precise mechanism of this effect should be investigated more in detail molecular level. 

In a previous study on human subjects, Cuban policosanol treatment unexpectedly reduced blood pressure in healthy male participants [9]. In order to confirm the blood pressure lowering effect of policosanol in a hypertension animal model, we supplemented policosanol to spontaneously hypertensive rats (SHR). 

SHR are the most widely used animal model to study hypertension accompanied by vascular oxidative stress [12]. Oxidative stress precedes a rise in blood pressure and contributes to the development of hypertension. We hypothesized that antioxidant treatment such as policosanol in SHR reduces oxidative stress to prevent the development of high blood pressure and improve the lipid profile. 

In order to confirm the blood pressure lowering effect of policosanol detail, the policosanol was supplemented to SHR (12-weeks-old) at three dosages (20, 100, and 200 mg/kg of body weight). The effects of policosanol on heart rate (HR), arterial blood pressure, serum lipid profile, and hepatic inflammation were also examined between the groups.

## 2. Results

### 2.1. Blood Pressure and Heart Rate

At week 0, all SHR groups showed similar ranges of SBP and DBP (around 187–190 mmHg and 144–151 mmHg, respectively) while normotensive Wistar Kyoto (WKY) rats showed normal ranges of SBP and DBP (around 117 mmHg and 89 mmHg, respectively). During the eight-week consumption period, the SHR control showed approximately 1.2-fold increases in SBP and DBP (around 228 mmHg and 178 mmHg respectively), as shown in Table 1. However, the policosanol groups showed reduction of blood pressure in a dose-dependent manner. Specifically, the 200 mg of policosanol group showed the lowest SBP and DBP at eight weeks up to sixteen percent reduction from its initial level (*p* < 0.001), whereas the 20 mg and 100 mg groups showed a four percent increase and five percent decrease, in SBP. DBP also increased by 17.6% in the SHR control group at eight weeks. However, 100 mg and 200 mg of policosanol consumption reduced DBP in a dose-dependent manner around 12–16% reduction compared to week 0. (Table 1).

**Figure 1:**
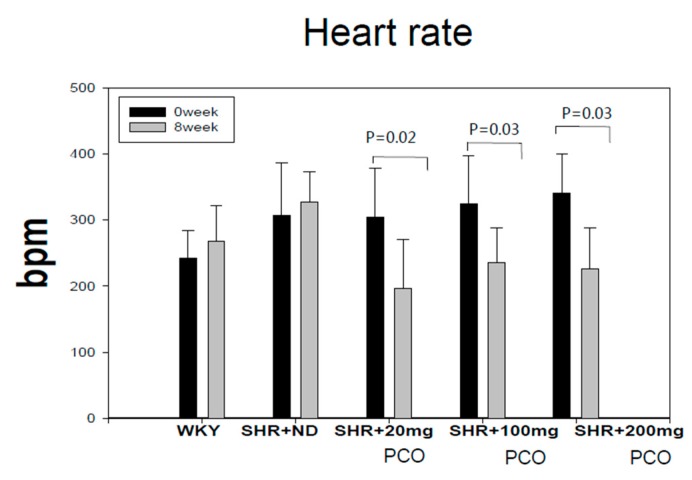
Changes in heart rate (HR) during eight weeks of policosanol supplementation. bpm, beats per minute; ND, normal diet; PCO, policosanol.

At week 0, all SHR groups had similar HRs (bpm) of around 305–341 bpm, whereas WKY rats had a HR of 242 ± 22 bpm. At week 8, the SHR + ND group had an even six percent higher HR, whereas the policosanol group had a reduced HR compared with that at week 0. The 20 mg of policosanol group showed a 36% reduction in HR at week 8, whereas the 100 mg and 200 mg of policosanol groups showed reduction of 28% and 34% respectively, at week 8 (Figure 1).

**Figure 2:**
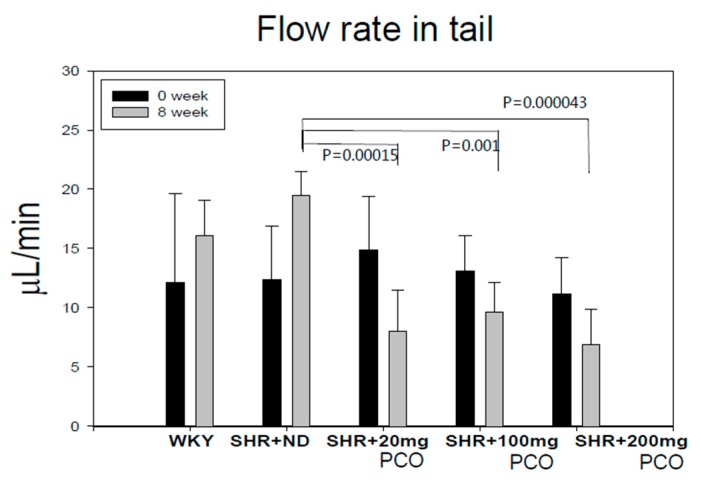
Changes in flow rate in tail during eight weeks of policosanol supplementation. ND, normal diet; PCO, policosanol.

As shown in Figure 2, tail flow rates at week 0 were similar among all groups around (11–15 µL/min). At week 8, tail flow rate increased 1.6-fold in the SHR + ND group in a time-dependent manner, whereas the policosanol groups showed significant reduced flow rates up to 30–47% in a dose-dependent manner. The SHR + 200 mg of policosanol group showed the slowest flow rate around (7 µL/min), whereas the SHR + ND group showed a flow rate of 20 µL/min.

**Figure 3:**
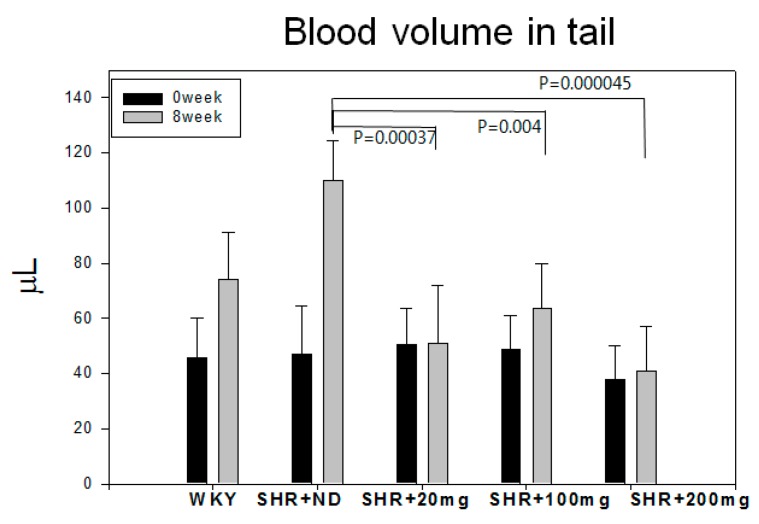
Changes in blood volume in tail eight weeks of policosanol supplementation. ND, normal diet; PCO, policosanol.

At week 0 blood volumes in the tail were similar among all groups (around 40–50 µL) (Figure 3). At week 8, WKY rats showed a 1.6-fold increase in tail blood volume compared with week 0, whereas the SHR + ND group showed the highest increase around 2.3-fold at week 8 up to 110 ± 21 µL. However, the policosanol groups showed reduction of tail blood volume. The 200 mg of policosanol group showed the lowest tail blood volume around 41 ± 10 µL with the highest significance (Figure 3).

### 2.2. Serum Lipid Profile

There was no difference in water and food consumption in all groups during the eight weeks. WKY rats showed a 1.8-fold increase in plasma TC level (around 133 mg/dL) than the SHR + ND group, and all SHR groups with or without policosanol consumption had similar TC level (around 73–76 mg/dL) at week 8. The SHR + ND group showed a 1.4-fold higher plasma TG level than the WKY group. Policosanol consumption reduced the TG level in a dose-dependent manner. The 200 mg of policosanol group showed the lowest TG level-up to 19% lower than that of the SHR + ND group. At week 8, as shown in Table 2, the SHR + ND group (44 mg/dL) showed a 35% lower HDL-C level than WKY rats (68 mg/dL). However, HDL-C level was elevated after policosanol consumption in a dose-dependent manner up to 52 mg/dL in the 100 mg and 200 mg of policosanol groups. The HDL-C/TC ratio (% HDL-C) also increased upon policosanol consumption up to 70% in the SHR + 200 mg of policosanol group. The SHR control group showed a two-fold higher % TG/HDL-C ratio than the WKY group. However, the TG/HDL-C ratio was significantly reduced (up to 22%) to approximately 1.5 ± 0.1 upon policosanol consumption in a dose-dependent manner in the SHR + 200 mg of policosanol group.

Serum GOT level was similar between the WKY and SHR control groups. However, the 20 mg of policosanol group showed a 33% reduction in GOT level, although the effect was not dose-dependent manner. Moreover, the GPT level was not significantly different between the WKY and SHR groups.

The SHR control group showed a 2.1-fold higher CRP level than the WKY group. However, the serum CRP level was significantly reduced upon policosanol consumption in a dose-dependent manner (Table 2). The 200 mg of policosanol group showed a 45% lower CRP level than the SHR control group.

### 2.3. Serum CETP Activity

**Figure 4:**
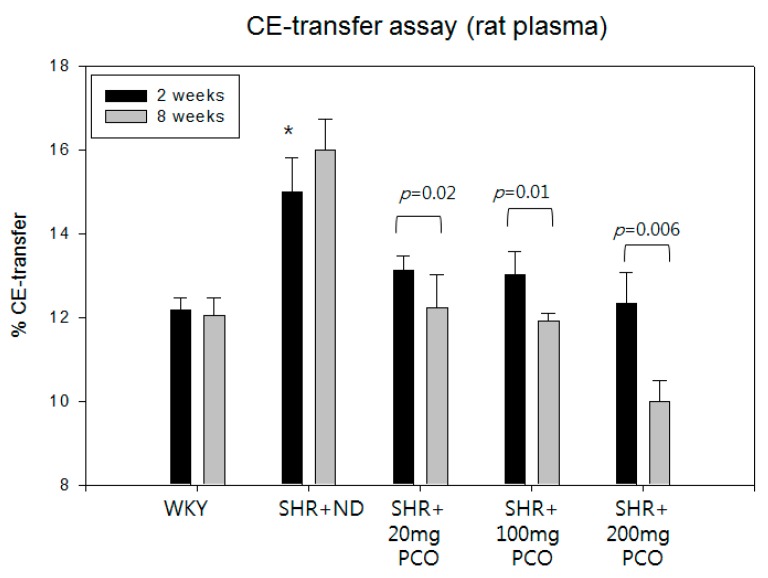
CE-transfer activity of WKY rats and SHR plasma after eight weeks of policosanol supplementation. Data shown are the mean ± SD of three independent experiments performed in duplicate. CE-transfer from [^3^H]-HDL (50 µg of apoA-I, 30,000 CPM) to human LDL (50 µg of protein) by rat plasma.

At week 2, the SHR control group showed higher CETP activity than WKY rats, as shown in Figure 4. At eight weeks, the SHR + ND group showed slightly increased CETP activity up to 16 ± 1% CE-transfer. However, CETP activity was reduced upon policosanol consumption in a dose-dependent manner at week 8. The 200 mg of policosanol group showed significant reduction of CETP activity up to 10 ± 0.5% CE-transfer as shown in Figure 4. The 20 mg and 100 mg of policosanol groups showed significant reductions of CETP activity at week 8 (12 ± 0.6% CE-transfer).

### 2.4. Antioxidative Activity of Lipoproteins

**Figure 5:**
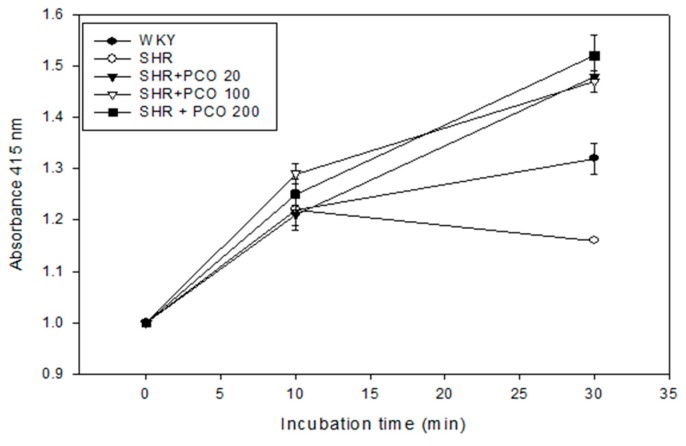
Changes of the paraoxonase activity in HDL after eight weeks of policosanol consumption in different groups.

After policosanol consumption, paraoxonase activity for HDL in the policosanol groups were elevated by 27.5%, 26.7%, and 31%, respectively, compared to the control group, as shown in Figure 5. This result implies that antioxidant activity was elevated in SHR group after consumption of policosanol.

### 2.5. Protein, TC, and TG Compositions of Lipoproteins

WKY rats showed higher total protein (TP) and total cholesterol (TC) contents in LDL, HDL_2_, and HDL_3_ fractions (Table 3). However, interestingly, the SHR control showed a higher TG content than the WKY group. In LDL fractions, the SHR + ND group showed the highest TP, TC, and TG levels. However, policosanol consumption caused gradual reductions of TC and TG levels in LDL in a dose-dependent manner. In HDL_2_, SHR showed only 45% lower TC and 21% higher TG levels than WKY rats. Policosanol consumption caused significant elevation of TC and reduction of TG levels. In HDL_3_, the SHR group showed 24% lower TC and 33% higher TG levels than the WKY group. However, policosanol consumption (200 mg of policosanol group) caused gradual elevation of TC in HDL_3_ up to 1.4-fold more elevation than SHR control group. Lastly, TG content gradually decreased in the policosanol groups in a dose-dependent manner.

### 2.6. Electrophoretic Profiles of LDL and HDL

**Figure 6:**
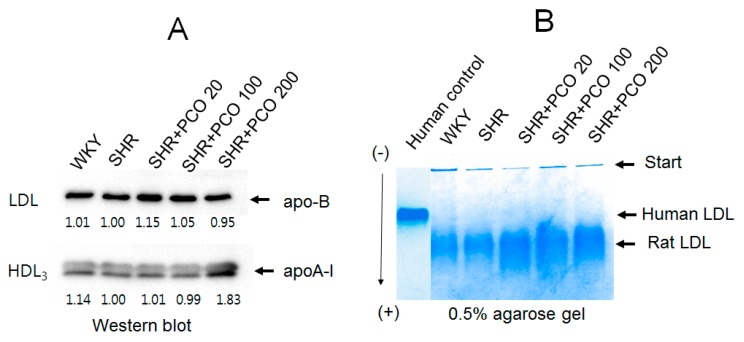
Electrophoretic profiles of LDL and HDL. (**A**) Immunodetection of LDL and HDL_3_ using anti-apoA-I and anti-apo-B antibodies. (**B**) Electromobility of LDL in agarose gel electrophoresis.

In the immunodetection experiment, apo-B levels in LDL were not significantly different between the groups, as shown in Figure 6A, regardless of policosanol consumption. However, apoA-I band intensity in HDL_3_ was elevated (up to 83%) in the 200 mg of policosanol group, whereas other policosanol groups showed similar levels as the control.

### 2.7. Electromobility of LDL

As shown in Figure 6B, LDL from WKY rats showed more aggregated band in the loading position, whereas human LDL showed a clear and thick band. However, LDL from SHR showed faster electromobility than human LDL with greater smear intensity. The electromobility of rat LDL was slower in policosanol consumption group in a dose-dependent manner.

### 2.8. Aldosterone Levels in the Different Study Groups

**Figure 7:**
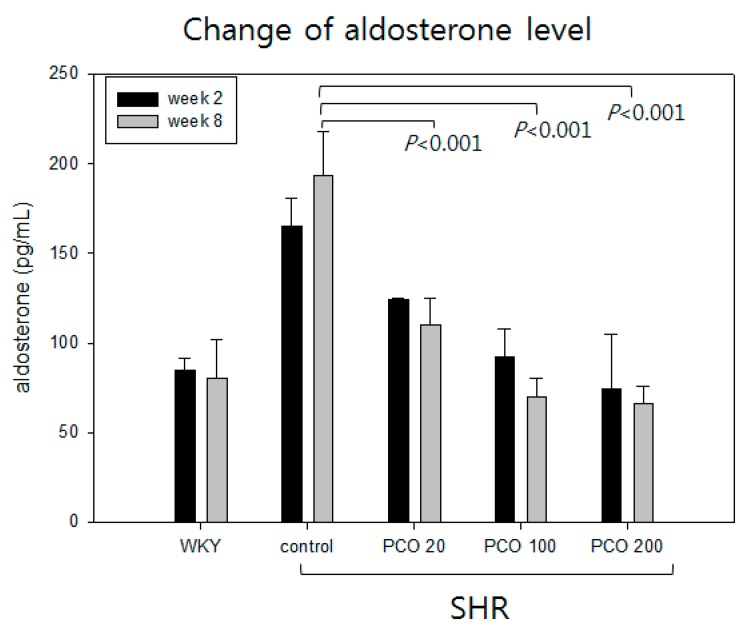
Changes in aldosterone level during eight weeks of policosanol consumption.

At week 2, the SHR control group showed a two-fold higher aldosterone level than the WKY group, as shown in Figure 7. At eight weeks, the SHR + ND (control) group showed a slight increment in the levels of aldosterone compared to week 0. However, aldosterone level decreased in the policosanol groups in a dose-dependent manner. At week 8, the 200 mg of policosanol group showed a serum aldosterone level of 66 ± 10 pg/mL whereas the SHR control group showed a level of 193 ± 25 pg/mL.

### 2.9. Histologic Analysis of Hepatic Tissue

**Figure 8:**
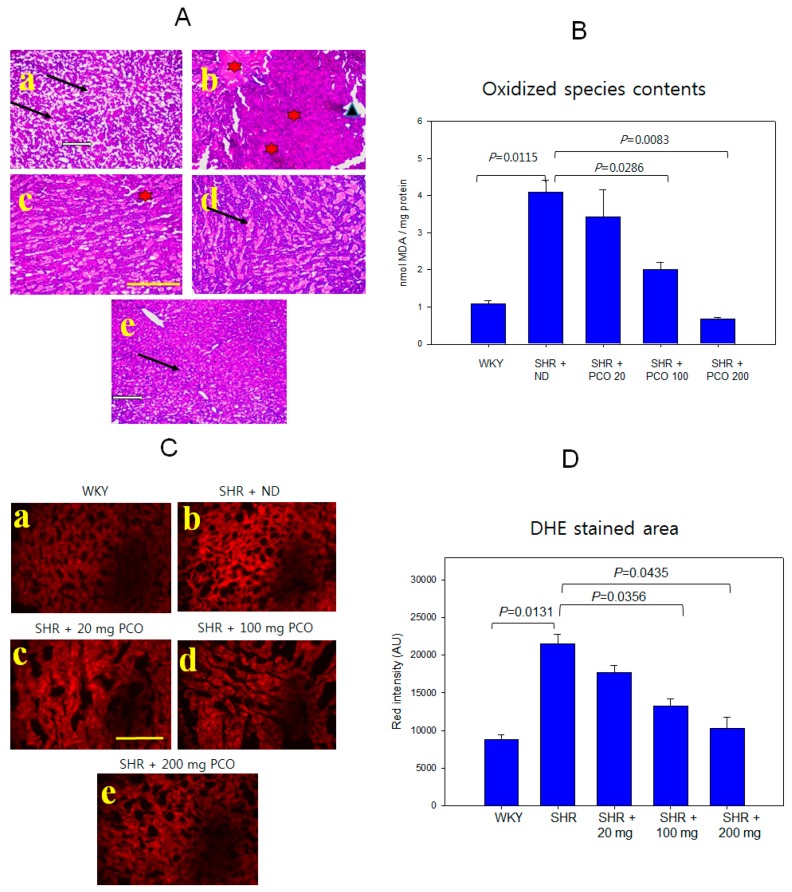
Liver histology by hematoxylin and eosin (H&E) and DHE staining. The bar in the photo indicates 100 μm (**A**) Representative image of histological changes in liver tissue observed by H&E staining in normotensive (WKY) rats and spontaneously hypertensive rats (SHR). Photo a, Normal WKY group showing normal hepatic lobulation and hepatocytes. Photo b, SHR showing loss of normal architecture of hepatocytes (indicated by a triangle) and presence of leukocytes infiltrations (indicated by star shape) and degenerative changes in hepatocytes. Photo c, SHR + 20 mg of policosanol (20 mg/kg of body weight). Photo d, SHR + 100 mg of policosanol (100 mg/kg of body weight). Photo c and d show potential improvement along with moderate reinstatement of hepatic lobule architecture and less infiltration of leukocytes. Photo e, SHR + 200 mg of policosanol (200 mg/kg of body weight) depicted a normal tissue appearance, marked improvement of minimal inflammatory infiltrate, preservation of classical lobular pattern, and normal shape of sinusoids (white arrow). Scale bar indicates 100 µm (Representative micrographs: magnification, ×100). (**B**) Quantification of oxidized species in hepatic tissue homogenate. (**C**) Visualization of ROS by DHE staining (Ex = 588 nm, Em = 615 nm). (**D**) Quantification of DHE-stained area. Quantification of fluorescence in embryos using computer-assisted morphometry within the same area (7.4 mm^2^). Data are shown as the mean ± SD of three independent experiments performed in duplicate. * *p* < 0.05; ** *p* < 0.01. The scale bar is 100 µm.

The WKY control group showed normal hepatic lobulation and hepatocytes, as shown in Figure 8A. The SHR control showed loss of normal architecture as well as the presence of macro- and microvesicular steatosis accompanied by inflammatory infiltrate and degenerative changes in hepatocytes. Lower doses of policosanol (20 mg and 100 mg) showed potential improvement along with moderate reinstatement of hepatic lobule architecture, moderate hepatocytes degeneration, and less infiltration of inflammatory cells, as indicated by the arrow in the figure. A higher dose of policosanol 200 mg showed marked improvement accompanied by minimal fibrotic changes and less infiltration of inflammatory cells. Most hepatocytes looked normal and showed minimal residual degenerative changes, as shown in Figure 8A.

Determination of oxidized species in the homogenate of hepatic tissue revealed that the SHR + ND group had the highest content of MDA, which was four-fold higher than that of WKY control group (Figure 8B). However, the policosanol groups showed a remarkable reduction of MDA content in a dose-dependent manner. The SHR + 200 mg of policosanol group contained 0.7 nmol of MDA, which was an 85% reduction compared with that of the SHR + ND group (4.1 nmol of MDA). The SHR + 100 mg of policosanol group showed a 50% reduction in MDA content (2.0 nmol) in liver extract, whereas the SHR + 20 mg of policosanol group did not show a significant reduction. DHE staining showed that ROS production was 2.4-fold higher in the SHR + ND group than the WKY group (Figure 8C). However, the policosanol groups showed 18%, 39%, and 53% reductions in DHE staining (Figure 8D). The 20 mg, 100 mg, and 200 mg of policosanol groups showed amelioration of hepatic inflammation and ROS production, as shown in Figure 8.

### 2.10. Correlation Analysis

The Pearson’s correlation analysis was performed mainly for the SHR + 100 mg and SHR + 200 mg of policosanol groups as shown in Supplementary Tables S1 and S2. The correlation study evaluated the relationship between blood pressure, HR, blood flow, and blood volume before and after eight weeks of policosanol treatment. The most significant correlation among these parameters was observed in the SHR + 200 mg of policosanol group. SBP and DBP were positively correlated with blood flow and blood volume after eight weeks of policosanol treatment. The HR was not found to be significant in either group (SHR + 100 mg of policosanol and SHR + 200 mg of policosanol groups).

## 3. Discussion

Hypertension is a major risk factor for the development of metabolic syndrome and CVD, and is associated with high oxidative stress and aortic stiffness. SHR are the most common animal model of human essential hypertension [13,14,15] accompanied by preceding elevation of arterial stiffness. Policosanol enhances the beneficial functions of HDL and maximizes its antioxidant, anti-glycation, and anti-atherosclerotic activities [6,7,9]. Although short-term consumption of policosanol has been shown to reduce blood pressure as well as increase HDL-C level and functionality in a human study, no study has yet examined essential hypertension using an SHR model with a focus on lipid/lipoprotein levels. 

As shown in Table 1, SBP and DBP of all SHR groups were not significantly different at week 0. However, policosanol supplementation potently reduced SBP and DBP in SHR at week 8 (Table 1). The SHR + ND group showed a 1.2-fold increase in SBP and DBP compared to week 0. At week 0, the SHR + ND group showed a 1.3-fold higher HR than WKY rats, as reported previously [16]. Elevation of HR was accompanied by an increase in blood pressure in SHR and HR was reduced by policosanol consumption, as shown in Figure 1. In support of our current results, Wu et al. reported that normal WKY rats had a HR of 381 ± 28 bpm, and SHR had a higher HR of 407 ± 26 bpm. SHR fed losartan, a classic selective AT1 receptor antagonist that inhibits myocardial fibrosis, showed reduction of HR as well as SBP and DBP [17]. 

The SHR groups showed lower blood TC and HDL-C levels than the WKY groups. Another report supports our current results, which shows that SHR had 15% lower HDL-C and 7% lower TC concentration [18]. Although SHR had a smaller body weight than WKY rats, SHR had a much higher serum TG level and TG/HDL-C ratio than WKY rats. These results might be associated with higher CETP activity in SHR groups. It is well known that higher CETP activity is correlated with higher serum TG levels [19] and myocardial infarction in patients [20]. Our recent report using cord serum from small neonates also found that small neonates had a two-fold higher TG content and 1.2-fold higher CETP activity [21]. To the best of our knowledge, this is the first report to measure CETP activity in SHR and WKY rats which is crucial to monitor atherogenic dyslipidemia, and atherosclerotic CVD. It has been known that mouse and rat are very resistant to develop atherosclerosis since they do not have CETP activity [22,23]. However, SHR show a significant time-dependent elevation of CETP activity compared to WKY rats, suggesting that higher CETP activity is associated with elevated blood pressure. In a previous human study, plasma CETP activity was shown to be inversely related to change in SBP, but not related to change in DBP [24]. Moreover, consumption of 600 mg of evacetrapib had no significant effects on either SBP or DBP compared with placebo [25]. 

In our previous report, policosanol in rHDL showed potent CETP inhibitory activity similar to that of anacetrapib [6,7], although policosanol showed less CETP inhibitory activity in ethanol. In the DEFINE Phase 3 safety study, anacetrapib was found to lower LDL-C and elevate HDL-C levels, whereas no changes in blood pressure or electrolyte or aldosterone levels [26]. 

Our current study reported that SHR treated with policosanol group after eight weeks recorded an elevation of paraoxonase activity in HDL, suggesting an enhancement in antioxidant capacity and functions. Policosanol could inhibit glycation of lipoproteins based on our previous reports [7,9,10], advanced glycation end product could make stiffness in the blood vessel which causes to raise blood pressure [27]. Raising HDL-C [28] and inhibition of CETP activity [29] was associated with improvement of serum lipid profile that resulted in lowering blood pressure.

**Figure 9:**
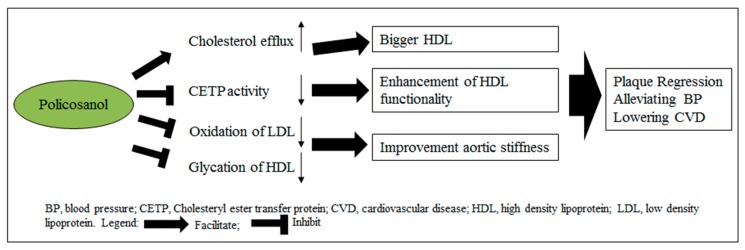
Proposed flow chart elucidating the mechanism of policosanol in lowering blood pressure.

Previous reports have suggested that elevated aldosterone levels could increase the risk of hypertension [30,31]. Moreover, basic and clinical studies have demonstrated that the role of renin– angiotensin–aldosterone system (RAAS) in hypertension [32,33]. Therefore, we examined the level of aldosterone in different groups (WKY, SHR, and SHR treated policosanol group) of animals. Our study reported a lowering of aldosterone in policosanol treated SHR group in a dose-dependent manner. This study proposed a mechanism for the blood pressure lowering effect of policosanol which is depicted in Figure 9. The inhibition of CETP activity, oxidation of LDL and glycation of HDL improves the aortic stiffness and thus lowers the blood pressure in SHR treated with policosanol.

From Pearson’s correlation analysis, SBP and DBP at week 0 were shown to be inversely correlated with tail blood flow rate and tail blood volume. However, at week 8, the relationship changed to be positive in a dose-dependent manner, especially in the SHR + 200 mg of policosanol group. Previously these parameters were used to monitor blood pressure in various models [34,35]. The serum CRP level was 2.1-fold higher in SHR than in WKY rats, which is in good agreement with others reports [36,37] and decreased upon policosanol consumption.

## 4. Materials and methods

### 4.1. Animals and Diets

Male spontaneously hypertensive rats (SHR/Izm; SHR, *n* = 50), 10-weeks-old, and age-matched Wistar Kyoto normotensive (WKY/Izm) rats were purchased from Japan SLC, Inc (Hamamatsu, Japan). Experiments were performed in accordance with the Guiding Principles for the Care and Use of Laboratory Animals approved (2016-011) by the Committee of Animal Welfare of Yeungnam University (Gyeongsan, Korea). Rats were maintained on a 12-h light/dark cycle and allowed access to food and water ad libitum. After 2 weeks of acclimation, all rats were supplemented with each designated diet as shown in Table 1; normal diet (ND) alone, ND + 20 mg, 100 mg, and 200 mg of policosanol per kg of body weight for SHR and WKY rats consumed ND alone.

Policosanol (sugar cane wax alcohol, SCWA) was obtained from Rainbow & Nature Pty, Ltd (Thornleigh, NSW, Australia). Policosanol consists of several alcohol chains of various lengths. Contents of the higher aliphatic alcohols were more than 90%. The individual alcohols present in policosanol are 1-tetracosanol (C_24_H_49_OH; molecular weight (MW): 354.7 mµ) ≤ 2%; 1-hexacosanol (C_26_H_53_OH; MW: 382.4 mµ) ≤ 4.5–10%; 1-heptacosanol (C_27_H_55_OH; MW: 396.4 mµ) ≤ 5%; 1-octacosanol (C_28_H_57_OH; MW: 410.5 mµ) ≤ 60–70%; 1-nonacosanol (C_29_H_59_OH; MW: 424.8 mµ) ≤ 2%; 1-triacontanol (C_30_H_61_OH; MW: 438.5 mµ) ≤ 10–15%; 1-dotriacontanol: (C_32_H_65_OH; MW: 466.5 mµ) ≤ 3–8%; 1-tetratriacontanol (C_34_H_69_OH; MW: 494.5 mµ) ≤ 2%. Policosanol was mixed with powder of purified rodent diet AIN-76A (Dyets, Inc, Bethlehem, PA, USA) to produce daily contents of 20, 100, and 200 mg of policosanol/kg of body weight. The mixtures were then re-pelleted and stored at 4 °C during the 8-weeks feeding period.

### 4.2. Measurement of Blood Pressure

Systolic blood pressure (SBP) and diastolic blood pressure (DBP) were measured using a CODA^TM^ monitor (Kent Scientific Co, Torrington, CT, USA), a computerized, non-invasive blood pressure monitor, according to the owner’s manual using the tail-cuff manometry. Heart rate (HR, bpm), blood volume (µL), and flow rate (mL/min) were also measured using the CODA^TM^ monitor equipped with a volume pressure recording sensor. Baseline blood pressure and HR measurements were recorded for 7 days before the initiation of policosanol consumption. During consumption, blood pressure was recorded at 2-week intervals after training the animals. The procedure of measuring blood pressure are as follow, unanesthetized rats were controlled in a plastic tube, that was positioned on a heating pad for the maintenance of the body temperature and to establish ample blood flow to the tails. Each rat was allowed to adjust to the restrainer for 5–7 min and then measure blood pressure. The volume-pressure recording determines the tail blood volume using a volume pressure recording sensor and an occlusion tail-cuff. It measures the SBP, DBP, heart rate, tail blood volume, and tail blood flow. SBP and DBP were recorded three times blind to the randomization sequence at each interval and average values were calculated. The CODA^TM^ monitor system includes a controller, laptop computer, software, cuffs, animal holders, infrared warming pads, and an infrared thermometer.

### 4.3. Plasma Analysis

After 8 weeks of policosanol intake, blood was obtained from rats by heart puncture following overnight fasting. During consumption, small blood samples were collected from each tail vein and stored in an Eppendorf tube containing EDTA at 2, 4 and 8 weeks. Plasma was separated by low-speed centrifugation (3000 *g*) and stored at −80 °C. TC, TG, HDL-C, glucose, GOT, and GPT levels were analyzed by using the available kits (Cleantech TS-S; Wako Pure Chemical, Osaka, Japan). LDL-cholesterol (LDL-C) was calculated using the Friedewald formula: LDL-C = TC − (HDL-C + (TG/5)).

### 4.4. Purification and Analysis of Lipoproteins

A sequential ultracentrifugation were used to separate very low-density lipoproteins (VLDL, d < 1.019 g/mL), LDL (1.019 < d < 1.063), HDL_2_ (1.063 < d < 1.125), and HDL_3_ (1.125 < d < 1.225) from the serum of each group [38]. Moreover, the density was adjusted by supplementation of NaCl and NaBr based on the standard protocols. Blood was centrifuged for 24 h at 10 °C at 100,000 *g* using a Himac CP100NX (Hitachi, Tokyo, Japan) using P50AT4-0124 rotor in our laboratory. 

The concentration of Protein from the lipoproteins were calculated by Lowry protein assay, further modified by Markwell et al. [39]. To quantify the oxidation of each LDL, the amount of oxidized species in LDL was measured by the thiobarbituric acid reactive substances (TBARS) method [40].

### 4.5. Oxidation of LDL by Cupric Ion

To measure the susceptibility of cupric ion-mediated LDL oxidation, 300 µg of LDL was nurtured with 10 µM CuSO_4_ for up to 3 h. The quantity of conjugated dienes formation was observed by measuring absorbance at 234 nm (Abs_234_) at 37 °C [41] using a Beckman DU 800 spectrophotometer. To corroborate the spectroscopic data, oxLDL samples were loaded on 0.5% agarose gel electrophoresis to differentiate relative electromobilities [42]. The electrophoretic mobility of each LDL was examined by 0.5% agarose gel electrophoresis. More oxidized LDL travel faster towards the bottom of the gel than less oxidized or native LDL, and electrical properties of oxLDL were shifted with a reduced size because of apo-B fragmentation.

### 4.6. Assay for Cholesteryl Ester Transfer Protein 

A rHDL-containing apoA-I and cholesteryl oleate were synthesized based on the method reported by Cho [43]. Briefly, lipids (POPC, cold cholesteryl oleate, and [^3^H]-cholesteryl oleate) were mixed in a glass vial and gently vortexed, followed by drying under a N_2_ gas stream at 37 °C. After drying, the lipids were dispersed by addition of TBS with slight agitation. Phospholipid bilayer formation was facilitated by the addition of sodium cholate and apoA-I. After extensive dialysis for 24 h to remove cholate, [^3^H]-CE-rHDL was recovered and characterized by scintillation counting and protein determination.

The [^3^H]-CE-rHDL was incapacitated using CNBr-activated Sepharose 4B resin from Amersham Biosciences for trouble-free separation after the reaction, according to the manufacturer's protocol. CE transfer reaction was done in 300-µL reaction mixtures consisting of human serum (20 µL) or HDL_3_ (20 µL, 2 mg/mL) as a potential source of CETP. Human LDL (20 µL, 0.25 mg/mL) acts as a CE-acceptor while [^3^H]-rHDL-agarose (20 μL, 0.25 mg/mL) as a CE-donor. After incubation of 4 h at 37 °C, the reaction was halted by a brief centrifugation (10,000 *g*) for 3 min at 4 °C. The supernatant containing CE-acceptor (150 µL) was used in scintillation counting for measuring the ionizing radiation and % transfer of [^3^H]-CE from [^3^H]-rHDL to LDL was quantified.

### 4.7. Paraoxonase Assay 

Paraoxonase-1 (PON-1) activity was determined by measuring the initial velocity of *p*-nitrophenol production at 37 °C based on its absorbance at 405 nm (microplate reader, Bio-Rad model 680; Bio-Rad, Hercules, CA, USA), as described previously [44] with slight modification [45]. Prior to the measurement, HDL was thoroughly dialyzed against PBS to eliminate EDTA.

### 4.8. ELISA and Western Blotting

To quantify aldosterone and C-reactive protein (CRP) in plasma, an aldosterone ELISA kit (ab136933) and rat CRP ELISA kit (PTX1, ab108827), respectively, were obtained from Abcam (Cambridge, UK). Appropriately diluted plasma samples were reacted using the kit in according to the manufacturer’s suggestion.

The composition of apolipoprotein/lipoprotein were analyzed by sodium dodecyl sulfate-polyacrylamide gel electrophoresis (SDS-PAGE) with an exact amount of protein loaded in the gel (5 µg of total protein per lane) from individual HDL_3_, and the levels of apolipoprotein expression were measured by immunodetection. Anti-human apoA-I antibody (ab7613) and anti-apo-B antibody (ab20737) were ordered from Abcam (Cambridge, UK). Relative band intensity (BI) was compared through band scanning with Chemi-Doc^®^ XRS+ (Bio-Rad, Hercules, CA, USA) using Image Lab software (Version 5.2, Bio-Rad, Hercules, CA, USA). Blot images are imported into the Quantity One software and then contrast was adjusted in a way so that the bands were fully noticeable on the blot image. The area around the band was chosen; additionally, the background intensity was deducted from the blot image. The appropriate bands were used to calculate band intensities and exported to excel for analysis. 

### 4.9. Histologic Analysis

The livers of the sacrificed rats were fixed in 4% paraformaldehyde for 24 h. Fixed tissues were then inserted in Tissue-Tek OCT compound (Thermo, Walldorf, Germany) and frozen. After frozen the tissue blocks were fixed in a model Leica CM1510S cryotome (Nussloch, Germany), and 7-μm sections of individual tissue were placed on 3-aminopropyltriethoxysilane-coated slides. Six to seven successive sectioned slides from each rat were stained with hematoxylin and eosin according to the standard protocols.

To compare the oxidative stress in tissues, ROS levels were imaged using dihydroethidium (DHE, cat # 37291; Sigma, St. Louis, MO, USA) as described previously [46]. The image was seen by fluorescence (Ex = 588 nm and Em = 615 nm) using a Nikon Eclipse TE2000 microscope (Tokyo, Japan). Image-Pro plus software (version 4.5.1.22; Media Cybernetics, Bethesda, MD, USA) were used for the quantification of section fluorescence.

Aliquots of liver tissue (50 mg of liver in 0.5 mL of PBS) from all groups were homogenized for 3 min (150 rpm) using a tissue homogenizer with a standard protocol (Euro-ST; Eurostar, IKA-WERKE, Staufen, Germany). After centrifugation (10,000 *g*) Bradford reagent was used for the protein determination; equally diluted supernatants (100 μg of protein in 0.05 mL) were used for quantification of oxidative species by the thiobarbituric acid reactive species (TBARS) method [40].

### 4.10. Data Analysis

The data of this study are expressed as the mean ± SD from at least three independent experiments with duplicate samples. For the rat study, multiple groups were compared using one-way analysis of variance (ANOVA) between the five groups using Scheffe test. Correlation analysis was carried out using Pearson’s test. Statistical analysis was performed by SPSS software program (version 23.0; SPSS, Inc., Chicago, IL, USA). A *p* value < 0.05 was considered statistically significant.

## 5. Conclusions

In conclusion, eight weeks of policosanol consumption by SHR resulted in remarkable reduction of blood pressure, serum aldosterone, and serum TG levels, elevation of HDL-C level and lipoprotein functionality, and improvement of hepatic inflammation.

## Figures and Tables

**Table 1:** Changes in SBP and DBP during eight weeks in the study groups

Groups	SBP (Mean ± SD) Week 0	SBP (Mean ± SD) Week 8	*p-*Value	DBP (Mean ± SD) Week 0	DBP (Mean ± SD) Week 8	*p-*Value
WKY (*n* = 10)	117.7 ± 11.1	127.6 ± 10.3 *	0.048	89.1 ± 7.8	82.9 ± 7.3 *	0.039
SHR + ND (*n* = 10)	187.6 ± 12.2	228.9 ± 5.3 **	<0.001	151.6 ± 13.7	178.3 ± 12.4 **	<0.001
SHR + 20 mg (*n* = 10)	188.7 ± 12.4	196.3 ± 12.8	0.08	144.7 ± 14.6	139.9 ± 21.2	0.331
SHR + 100 mg (*n* = 10)	189.0 ± 11.5	180.6 ± 17.5	0.293	145.0 ± 10.1	126.3 ± 19.1	0.06
SHR + 200 mg (*n* = 10)	190.6 ± 18.7	160.3 ± 8.30 *	0.03	148.4 ± 12.7	124.6 ± 40.8 *	0.01

SBP, systolic blood pressure; DBP, diastolic blood pressure; WKY, normotensive Wistar Kyoto; SHR, Spontaneously hypertensive rats. *p*-Values calculated from the paired *t*-test at week 0 and week 8 of therapy. ** *p* < 0.001, compared with week 0, * *p* < 0.05 compared with week 0.

**Table 2:** Body weight changes and biochemical parameters in the study animals at week 8 ^1^.

Group	1	2	3	4	5
	WKY (*n* =10)	SHR + ND (*n* = 10)	SHR + 20mg (*n* = 10)	SHR + 100mg (*n* = 10)	SHR + 200mg (*n* = 10)
Body weight					
week 0	313 ± 10	288 ± 8	294 ± 7	291 ± 7	293 ± 13
week 8	409 ± 10	378 ± 8	384 ± 7	375 ± 6	374 ± 7
TC (mg/dL)	133 ± 3 ^¶¶¶^	75 ± 3	75 ± 4	73 ± 3	74 ± 5
TG (mg/dL)	61 ± 4 ^¶^	83 ± 7	89 ± 8	77 ± 9	68 ± 3 *
HDL-C (mg/dL)	67 ± 6 ^¶¶^	44 ± 3	44 ± 4	52 ± 4 *	52 ± 4 *
% HDL-C	48 ± 4	58 ± 3	60 ± 5	69 ± 3 *	70 ± 3 *
TG/HDL-C	0.9 ± 0.1 ^¶¶¶^	1.9 ± 0.1	2.1 ± 0.2	1.6 ± 0.1 **	1.5 ± 0.1 *
LDL-C ^2^	58 ± 4 ^¶¶¶^	14 ± 2.3	12 ± 4.6	8 ± 3.2 *	7 ± 1.8 *
Glucose (mg/dL)	131 ± 15	161 ± 22	149 ± 25	153 ± 23	162 ± 14
GOT (U/L)	25 ± 3	28 ± 3	19 ± 2	30 ± 4	30 ± 4
GPT (U/L)	20 ± 0.3	20 ± 0.5	21 ± 0.4	20 ± 0.4	21 ± 0.4
CRP (ng/L)	476 ± 25 ^¶¶^	988 ± 48	670 ± 40 *	660 ± 32 *	546 ± 29 **

^1^ Data are expressed as mean ± SD. Significance was obtained from independent samples t-test. ^¶¶¶^, *p* < 0.001 vs. SHR + ND; ^¶¶^, *p* < 0.01 vs. SHR + ND; ^¶^, *p* < 0.05 vs. SHR + ND, *** *p* < 0.001 vs. SHR + ND; ** *p* < 0.01 vs. SHR + ND; * *p* < 0.05 vs. SHR + ND. ^2^ LDL-C, calculated from Friedewald formula: LDL-C = TC-(HDL-C + (TG/5)). CRP, C-reactive protein; HDL-C, high-density lipoprotein cholesterol; LDL-C, low-density lipoprotein cholesterol; TC, total cholesterol; TG, triglycerides; GOT, glutamate oxaloacetate transaminase; GPT, glutamate pyruvate transaminase.

**Table 3:** Lipid and protein compositions in lipoprotein profile at week 8.

Group		1	2	3	4	5
		WKY (*n* = 10)	SHR + ND (*n* = 10)	SHR + 20 mg (*n* = 10)	SHR + 100 mg (*n* = 10)	SHR + 200 mg (*n* = 10)
LDL	TP (mg/mL)	1.6 ± 0.1	1.3 ± 0.1	1.1 ± 0.1	1.0 ± 0.1	0.8 ± 0.1 *
	TC (mg/mg)	11.4 ± 2.1	9.3 ± 0.8	8.4 ± 0.3	7.5 ± 0.4 *	7.0 ± 0.2 *
	TG (mg/mg)	3.1 ± 0.1	4.8 ± 0.1	4.4 ± 0.1 *	3.2 ± 0.0 **	3.0 ± 0.0 **
HDL_2_	TP (mg/mL)	2.8 ± 0.2	2.3 ± 0.2	2.2 ± 0.2	2.3 ± 0.2	3.2 ± 0.2 *
	TC (mg/mg)	12.0 ± 0.5	6.6 ± 0.2	10.2 ± 0.2 **	10.6 ± 0.5**	11.0 ± 0.2 **
	TG (mg/mg)	1.4 ± 0.1	1.7 ± 0.1	1.7 ± 0.2	1.5 ± 0.1	1.4 ± 0.1 *
HDL_3_	TP (mg/mL)	2.9 ± 0.2	2.7 ± 0.2	2.4 ± 0.1	2.6 ± 0.1	3.3 ± 0.2 *
	TC (mg/mg)	4.2 ± 0.1	3.2 ± 0.1	4.1 ± 0.1 **	4.3 ± 0.2 **	4.5 ± 0.2 **
	TG (mg/mg)	0.9 ± 0.0	1.2 ± 0.1	1.1 ± 0.0	0.9 ± 0.0 *	0.8 ± 0.0 **

Data are expressed as mean ± SD. WKY, normotensive Wistar Kyoto; SHR, Spontaneously hypertensive rats. HDL, high-density lipoproteins; LDL-C, low-density lipoprotein cholesterol; TC, total cholesterol; TG, triglyceride; TP, total protein. *** *p* < 0.001 vs. SHR + ND; ** *p* < 0.01 vs. SHR + ND; * *p* < 0.05 vs. SHR + ND in each group.

## Supplementary Materials

The following are available online.
