# Correction: Cho, K.H., et al. Blood pressure Lowering Effect of Cuban Policosanol is Accompanied by Improvement of Hepatic Inflammation, Lipoprotein profile, and HDL Quality in Spontaneously Hypertensive Rats. *Molecules* 2018, *23*, 1080

**DOI:** 10.3390/molecules24010194

**Published:** 2019-01-07

**Authors:** Kyung-Hyun Cho, Dhananjay Yadav, Suk-Jeong Kim, Jae-Ryong Kim

**Affiliations:** 1 Department of Medical Biotechnology, Yeungnam University, Gyeongsan 712-749, Korea; dhanyadav16481@gmail.com (D.Y.); superiorgene@ynu.ac.kr (S.-J.K.); 2 Research Institute of Protein Sensor, Yeungnam University, Gyeongsan 712-749, Korea; 3 LipoLab, Daehak-Ro 280, Gyeongsan 712-749, Korea; 4 Department of Biochemistry and Molecular Biology, Smart-Aging Convergence Research Center, College of Medicine, Yeungnam University, Daegu 705-717, Korea; kimjr000@gmail.com

The authors wish to make the following correction to their paper [1]. In Table 1, the data for SHR + 200 mg were incorrectly displayed. The correct version of the table is as follows: 

The change does not affect the scientific outcome. The manuscript will be updated and the original will remain online on the article webpage. The authors would like to apologize for any inconvenience caused to the readers by these changes.

## Figures and Tables

**Table 1:** Changes in SBP and DBP during eight weeks in the study groups.

Groups	SBP (Mean ± SD) week 0	SBP (Mean ± SD) week 8	*p*-Value	DBP (Mean ± SD) week 0	DBP (Mean ± SD) week 8	*p*-Value
WKY (*n* = 10)	117.7 ± 11.1	127.6 ± 10.3 *	0.048	89.1 ± 7.8	82.9 ± 7.3 *	0.039
SHR + ND (*n* = 10)	187.6 ± 12.2	228.9 ± 5.3 **	<0.001	151.6 ± 13.7	178.3 ± 12.4 **	<0.001
SHR + 20mg (*n* = 10)	188.7 ± 12.4	196.3 ± 12.8	0.08	144.7 ± 14.6	139.9 ± 21.2	0.331
SHR + 100mg (*n* = 10)	189.0 ± 11.5	180.6 ± 17.5	0.293	145.0 ± 10.1	126.3 ± 19.1	0.06
SHR + 200mg (*n* = 6)	190.3 ± 17.3	160.3 ± 20.3 *	0.03	148.8 ± 8.3	124.5 ± 33.5 *	0.01

SBP, systolic blood pressure; DBP, diastolic blood pressure; WKY, normotensive Wistar Kyoto; SHR, Spontaneously hypertensive rats. *p*-values calculated from the paired *t*-test at week 0 and week 8 of therapy. ** *p* < 0.001, compared with week 0, * *p* < 0.05 compared with week 0.
