# Supplementary material for: Blood Pressure Lowering Effect of Cuban Policosanol is Accompanied by Improvement of Hepatic Inflammation, Lipoprotein Profile, and HDL Quality in Spontaneously Hypertensive Rats
Source: Molecules. 2018 May 3;23(5):1080. doi: 10.3390/molecules23051080 (PMC6102548; doi:10.3390/molecules23051080)
Supplement: Supplementary file 1 [file molecules-23-01080-s001.pdf]

Supplementary Table 1 (S1). Pearson's correlation analysis between week 0 and 8 in SHR+100 mg of policosanol group.

| Group<br>SHR+100mg (n=10) | Data A | Data B | Correlation coefficient (r) | Significance. |
|---|---|---|---|---|
| Before (0week) | Systolic | Heart rate | 0.597 | 0.126 |
| After (8week) | | (bpm) | 0.680 | 0.063 |
| Before (0week) | Systolic | Blood flow | -0.428 | 0.290 |
| After (8week) | | (μL/min) | 0.615 | 0.059 |
| Before (0week) | Systolic | Blood volume | -0.685 | 0.061 |
| After (8week) | | (μL) | 0.741 | **0.014*** |
| Before (0week) | Diastolic | Heart rate | -0.239 | 0.569 |
| After (8week) | | (bpm) | 0.506 | 0.201 |
| Before (0week) | Diastolic | Blood flow | -0.411 | 0.312 |
| After (8week) | | (μL/min) | 0.775 | **0.009**** |
| Before (0week) | Diastolic | Blood volume | -0.616 | 0.104 |
| After (8week) | | (μL) | 0.850 | **0.002**** |

***, P<0.001; **, P<0.01, *, P<0.05

Supplementary Table 2 (S2). Pearson's correlation analysis between week 0 and 8 in SHR+200 mg of policosanol group.

| Group<br>SHR+200mg (n=10) | Data A | Data B | Correlation coefficient (r) | Significance. |
|---|---|---|---|---|
| Before(0week) | Systolic | Heart rate | -0.046 | 0.914 |
| After(8week) | | (bpm) | 0.447 | 0.267 |
| Before(0week) | Systolic | Blood flow | -0.123 | 0.753 |
| After(8week) | | (μL/min) | 0.874 | **0.001**** |
| Before(0week) | Systolic | Blood volume | -0.233 | 0.546 |
| After(8week) | | (μL) | 0.855 | **0.002**** |
| Before(0week) | Diastolic | Heart rate | -0.118 | 0.781 |
| After(8week) | | (bpm) | 0.623 | 0.099 |
| Before(0week) | Diastolic | Blood flow | -0.352 | 0.353 |
| After(8week) | | (μL/min) | 0.762 | **0.01*** |
| Before(0week) | Diastolic | Blood volume | -0.478 | 0.193 |
| After(8week) | | (μL) | 0.748 | **0.013*** |

***, *P<0.001*; **, *P<0.01*, *, *P<0.05*
